# Polarized blazar X-rays imply particle acceleration in shocks

**DOI:** 10.1038/s41586-022-05338-0

**Published:** 2022-11-23

**Authors:** Ioannis Liodakis, Alan P. Marscher, Iván Agudo, Andrei V. Berdyugin, Maria I. Bernardos, Giacomo Bonnoli, George A. Borman, Carolina Casadio, Víctor Casanova, Elisabetta Cavazzuti, Nicole Rodriguez Cavero, Laura Di Gesu, Niccoló Di Lalla, Immacolata Donnarumma, Steven R. Ehlert, Manel Errando, Juan Escudero, Maya García-Comas, Beatriz Agís-González, César Husillos, Jenni Jormanainen, Svetlana G. Jorstad, Masato Kagitani, Evgenia N. Kopatskaya, Vadim Kravtsov, Henric Krawczynski, Elina Lindfors, Elena G. Larionova, Grzegorz M. Madejski, Frédéric Marin, Alessandro Marchini, Herman L. Marshall, Daria A. Morozova, Francesco Massaro, Joseph R. Masiero, Dimitri Mawet, Riccardo Middei, Maxwell A. Millar-Blanchaer, Ioannis Myserlis, Michela Negro, Kari Nilsson, Stephen L. O’Dell, Nicola Omodei, Luigi Pacciani, Alessandro Paggi, Georgia V. Panopoulou, Abel L. Peirson, Matteo Perri, Pierre-Olivier Petrucci, Juri Poutanen, Simonetta Puccetti, Roger W. Romani, Takeshi Sakanoi, Sergey S. Savchenko, Alfredo Sota, Fabrizio Tavecchio, Samaporn Tinyanont, Andrey A. Vasilyev, Zachary R. Weaver, Alexey V. Zhovtan, Lucio A. Antonelli, Matteo Bachetti, Luca Baldini, Wayne H. Baumgartner, Ronaldo Bellazzini, Stefano Bianchi, Stephen D. Bongiorno, Raffaella Bonino, Alessandro Brez, Niccoló Bucciantini, Fiamma Capitanio, Simone Castellano, Stefano Ciprini, Enrico Costa, Alessandra De Rosa, Ettore Del Monte, Alessandro Di Marco, Victor Doroshenko, Michal Dovčiak, Teruaki Enoto, Yuri Evangelista, Sergio Fabiani, Riccardo Ferrazzoli, Javier A. Garcia, Shuichi Gunji, Kiyoshi Hayashida, Jeremy Heyl, Wataru Iwakiri, Vladimir Karas, Takao Kitaguchi, Jeffery J. Kolodziejczak, Fabio La Monaca, Luca Latronico, Simone Maldera, Alberto Manfreda, Andrea Marinucci, Giorgio Matt, Ikuyuki Mitsuishi, Tsunefumi Mizuno, Fabio Muleri, Stephen C.-Y. Ng, Chiara Oppedisano, Alessandro Papitto, George G. Pavlov, Melissa Pesce-Rollins, Maura Pilia, Andrea Possenti, Brian D. Ramsey, John Rankin, Ajay Ratheesh, Carmelo Sgró, Patrick Slane, Paolo Soffitta, Gloria Spandre, Toru Tamagawa, Roberto Taverna, Yuzuru Tawara, Allyn F. Tennant, Nicolas E. Thomas, Francesco Tombesi, Alessio Trois, Sergey Tsygankov, Roberto Turolla, Jacco Vink, Martin C. Weisskopf, Kinwah Wu, Fei Xie, Silvia Zane

**Affiliations:** 1 Finnish Centre for Astronomy with ESO, FI-20014, University of Turku, Turku, Finland; 2 Institute for Astrophysical Research, Boston University, Boston, MA USA; 3 Instituto de Astrofísica de Andalucía, IAA-CSIC, Glorieta de la Astronomía s/n, Granada, Spain; 4 Department of Physics and Astronomy, University of Turku, Turku, Finland; 5 INAF Osservatorio Astronomico di Brera, Merate (LC), Italy; 6 Crimean Astrophysical Observatory RAS, P/O Nauchny, Nauchnij, Crimea; 7 Institute of Astrophysics, Foundation for Research and Technology - Hellas, Voutes, Heraklion, Greece; 8 Department of Physics, University of Crete, Heraklion, Greece; 9 Agenzia Spaziale Italiana, Via del Politecnico snc, Roma, Italy; 10 Physics Department and McDonnell Center for the Space Sciences, Washington University in St Louis, St Louis, MO USA; 11 Department of Physics and Kavli Institute for Particle Astrophysics and Cosmology, Stanford University, Stanford, CA USA; 12 NASA Marshall Space Flight Center, Huntsville, AL USA; 13 Laboratory of Observational Astrophysics, St Petersburg University, St Petersburg, Russia; 14 Graduate School of Sciences, Tohoku University, Sendai, Japan; 15 Astronomical Institute, St Petersburg State University, St Petersburg, Russia; 16 Kavli Institute for Particle Astrophysics and Cosmology, Stanford University and SLAC, Menlo Park, CA USA; 17 Université de Strasbourg, CNRS, Observatoire Astronomique de Strasbourg, UMR 7550, Strasbourg, France; 18 Department of Physical Sciences, Earth and Environment, Astronomical Observatory, University of Siena, Siena, Italy; 19 MIT Kavli Institute for Astrophysics and Space Research, Massachusetts Institute of Technology, Cambridge, MA USA; 20 Istituto Nazionale di Fisica Nucleare, , Sezione di Torino, Torino, Italy; 21 Dipartimento di Fisica, Universitá degli Studi di Torino, Torino, Italy; 22 Caltech/IPAC, Pasadena, CA USA; 23 California Institute of Technology, Pasadena, CA USA; 24 Space Science Data Center, Agenzia Spaziale Italiana, Roma, Italy; 25 INAF Osservatorio Astronomico di Roma, Monte Porzio Catone (RM), Italy; 26 University of California, Santa Barbara, CA USA; 27 Institut de Radioastronomie Millimétrique, Granada, Spain; 28 Center for Research and Exploration in Space Science and Technology (CRESST), Greenbelt, MD USA; 29 Department of Physics and Center for Space Sciences and Technology, University of Maryland Baltimore County, Baltimore, MD USA; 30 INAF Istituto di Astrofisica e Planetologia Spaziali, Roma, Italy; 31 INAF-Osservatorio Astrofisico di Torino, Pino Torinese, Italy; 32 Université Grenoble Alpes, CNRS, IPAG, Grenoble, France; 33 Space Research Institute of the Russian Academy of Sciences, Moscow, Russia; 34 Special Astrophysical Observatory, Russian Academy of Sciences, Nizhnii Arkhyz, Russia; 35 Pulkovo Observatory, St Petersburg, Russia; 36 University of California Santa Cruz, Santa Cruz, CA USA; 37 INAF Osservatorio Astronomico di Cagliari, Selargius (CA), Italy; 38 Istituto Nazionale di Fisica Nucleare, Sezione di Pisa, Pisa, Italy; 39 Dipartimento di Fisica, Universitá di Pisa, Pisa, Italy; 40 Dipartimento di Matematica e Fisica, Universitá degli Studi Roma Tre, Roma, Italy; 41 INAF Osservatorio Astrofisico di Arcetri, Firenze, Italy; 42 Dipartimento di Fisica e Astronomia, Universitá degli Studi di Firenze, Sesto Fiorentino (FI), Italy; 43 Q30265285 Istituto Nazionale di Fisica Nucleare, Sezione di Firenze, Sesto Fiorentino (FI), Italy; 44 Istituto Nazionale di Fisica Nucleare, Sezione di Roma Tor Vergata, Roma, Italy; 45 Institut für Astronomie und Astrophysik, Tübingen, Germany; 46 Astronomical Institute of the Czech Academy of Sciences, Ondřejov, Czech Republic; 47 RIKEN Cluster for Pioneering Research, Saitama, Japan; 48 Yamagata University, Yamagata-shi, Japan; 49 Osaka University, Osaka, Japan; 50 University of British Columbia, Vancouver, British Columbia Canada; 51 Department of Physics, Faculty of Science and Engineering, Chuo University, Tokyo, Japan; 52 Graduate School of Science, Division of Particle and Astrophysical Science, Nagoya University, Nagoya, Japan; 53 Hiroshima Astrophysical Science Center, Hiroshima University, Hiroshima, Japan; 54 Department of Physics, University of Hong Kong, Pokfulam, Hong Kong; 55 Department of Astronomy and Astrophysics, Pennsylvania State University, University Park, PA USA; 56 Center for Astrophysics, Harvard & Smithsonian, Cambridge, MA USA; 57 Dipartimento di Fisica e Astronomia, Universitá degli Studi di Padova, Padova, Italy; 58 Dipartimento di Fisica, Universitá degli Studi di Roma Tor Vergata, Roma, Italy; 59 Mullard Space Science Laboratory, University College London, Dorking, UK; 60 Anton Pannekoek Institute for Astronomy & GRAPPA, University of Amsterdam, Amsterdam, the Netherlands; 61 Guangxi Key Laboratory for Relativistic Astrophysics, School of Physical Science and Technology, Guangxi University, Nanning, China

**Keywords:** High-energy astrophysics, Particle astrophysics

## Abstract

Most of the light from blazars, active galactic nuclei with jets of magnetized plasma that point nearly along the line of sight, is produced by high-energy particles, up to around 1 TeV. Although the jets are known to be ultimately powered by a supermassive black hole, how the particles are accelerated to such high energies has been an unanswered question. The process must be related to the magnetic field, which can be probed by observations of the polarization of light from the jets. Measurements of the radio to optical polarization—the only range available until now—probe extended regions of the jet containing particles that left the acceleration site days to years earlier^1–3^, and hence do not directly explore the acceleration mechanism, as could X-ray measurements. Here we report the detection of X-ray polarization from the blazar Markarian 501 (Mrk 501). We measure an X-ray linear polarization degree *Π*_X_ of around 10%, which is a factor of around 2 higher than the value at optical wavelengths, with a polarization angle parallel to the radio jet. This points to a shock front as the source of particle acceleration and also implies that the plasma becomes increasingly turbulent with distance from the shock.

## Abstract (web summary)

Polarization measurements are reported for the blazar Mk501, revealing a degree of X-ray polarization that is more than twice the optical value and supporting the shock-accelerated energy-stratified electron population scenario.

## Main

In blazars whose lower-energy emission component peaks in the X-ray band, such as Mrk 501, synchrotron radiation is the dominant emission process from radio to X-rays. Radiation at longer wavelengths probably arises from larger regions in the jet, and hence multiwavelength studies probe spatial variations in the magnetic field structure and other physical properties in different locations^4,5^. A particularly important diagnostic is the degree of order of the magnetic field and its mean direction relative to the jet axis, which can be determined by measurements of the linear polarization. For example, particle acceleration at a shock front should result in relatively high levels (tens of per cent) of X-ray linear polarization along a position angle that is parallel to the jet^6^. By contrast, more stochastic acceleration processes involving turbulence or plasma instabilities are expected to lead to weak polarization with random position angles. The optical, infrared and radio polarization probe the level of order and mean direction of the magnetic field in regions progressively farther from the site of particle acceleration. Simultaneous multiwavelength polarization from X-ray to radio, which is now achievable with the advent of the Imaging X-ray Polarimetry Explorer (IXPE^7^), can therefore provide a more complete picture of the emission region of a blazar jet than has previously been possible.

Variations in the flux of blazars at all wavebands, and in the linear polarization at radio to optical wavelengths, is largely stochastic in nature, which can be interpreted as the result of turbulence^5,6,8,9^. Multizone emission models, often involving a turbulent magnetic field, can reproduce a number of the observed characteristics of the variable linear polarization. In a turbulent region, roughly modelled as *N* cells, each with a uniform but randomly oriented field, we expect a mean degree of polarization of <*Π*> ≈ 75/√*N*, with the value of *Π* exhibiting variability on short timescales with a standard deviation of around 0.5<*Π*> (ref. ^5^), as often observed^10^. For a turbulent field in the plasma crossing a shock front, particle acceleration should be most efficient in cells for which the magnetic field is nearly parallel to the shock normal; this bias leads to a higher value of *Π* and more pronounced variability at X-rays compared to lower frequencies^5^. The passage of turbulent cells through the emission region would also cause irregular variations, including some apparent rotations, in the polarization angle (*ψ*)^8,11^.

On the other hand, some of the observed radio and optical patterns of polarization variability (for example, the above-mentioned *ψ* rotations) have been found to be inconsistent with purely stochastic processes^12,13^. This indicates that there is some coherent ordering of the magnetic field, for example, by compression or amplification by plasma processes in shocks^14^ or by the presence of a global, perhaps helical, magnetic field component^15–17^. In the commonly used single-zone model, the radiating particles are accelerated by an unspecified process to highly relativistic energies while being confined within a plasmoid with a partially ordered or helical magnetic field. The global magnetic field structure is expected to produce similar polarization patterns across frequencies, with little variability over time^18^. If the field is helical, *ψ* should align with the jet direction for most viewing angles^15^. In an alternative scenario, which includes shock acceleration, particles become energized over a limited volume, for example at a shock front, and then advect or diffuse away from that region^4,6,19^. In this process, the electrons lose energy to radiation, and so emit at progressively decreasing frequencies as they travel away from the acceleration site. We refer to this model as being ‘energy-stratified’. If the magnetic field is well ordered over the small volume of the acceleration region and becomes increasingly turbulent farther downstream, *Π* will decrease towards longer wavelengths, whereas *ψ* can vary with frequency if the mean direction of the magnetic field changes as the volume increases. In Mrk 501, we expect a progressively higher *Π* from radio to X-rays. A shock partially orders the magnetic field of the plasma crossing the shock, with the ordered field perpendicular to the shock normal. This causes the net polarization electric vector to be aligned with the jet. In a kink-instability-induced magnetic reconnection scenario, in which contiguous regions of oppositely directed magnetic field come into contact, the jet flow is sheared because of transverse velocity gradients^20^. Shearing would stretch the magnetic field along the jet boundary, so that *ψ* is expected to be transverse to the jet direction. The simultaneous contribution of multiple current sheets will lead to an overall lower polarization than in a shock scenario, with similar levels of polarization across frequencies^21^. Our expectations from the different emission models are summarized in Table 1.

**Table 1:** Summary of model properties

Model	Multiwavelength polarization	X-ray polarization variability^a^	X-ray polarization angle
Single zone	Constant^b^	Slow	Any
Multizone	Mildly chromatic	High	Any
Energy stratified (shock)	Strongly chromatic	Slow	Along the jet axis
Magnetic reconnection (kink instability)	Constant	Moderate	Perpendicular to the jet axis
Observed	Strongly chromatic	Slow	Along the jet axis

First, we find an increasing *Π* towards higher frequencies. Second, we do not find significant variability during the 2–3-day-long IXPE observations, and finally, we find a rough alignment of *ψ* with the jet axis from radio to X-rays. Therefore, a shock-accelerated, energy-stratified electron population model satisfies all our multiwavelength polarization observations.

^a^Slow variability, a few days to a week; moderate variability, days; high variability, less than 1 day.

^b^There is a slight dependence on the slope of the emission spectrum.

**Fig. 1:**
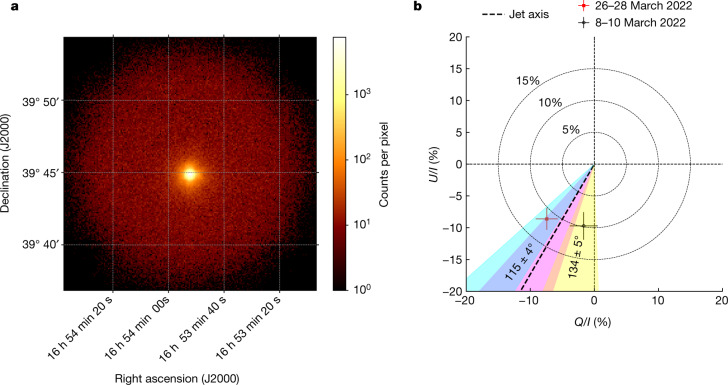
IXPE observations of Mrk 501. **a**, IXPE image of Mrk 501 during the 8–10 March 2022 observation in the 2–8 keV band. The colour bar denotes the number of X-ray photons per pixel. **b**, Normalized Stokes *Q*/*I* and Stokes *U*/*I* parameters, where *I* is the total intensity, of both IXPE observations. The yellow and cyan shaded regions denote the uncertainty (68% confidence interval (CI)) in the polarization angle for the 8–10 March and 26–28 March observations, respectively. The dashed black line shows the jet direction and the magenta shaded area its uncertainty (68% CI). The dashed circles mark different levels of polarization degree, as labelled. Error bars denote the 68% CI.

**Fig. 2:**
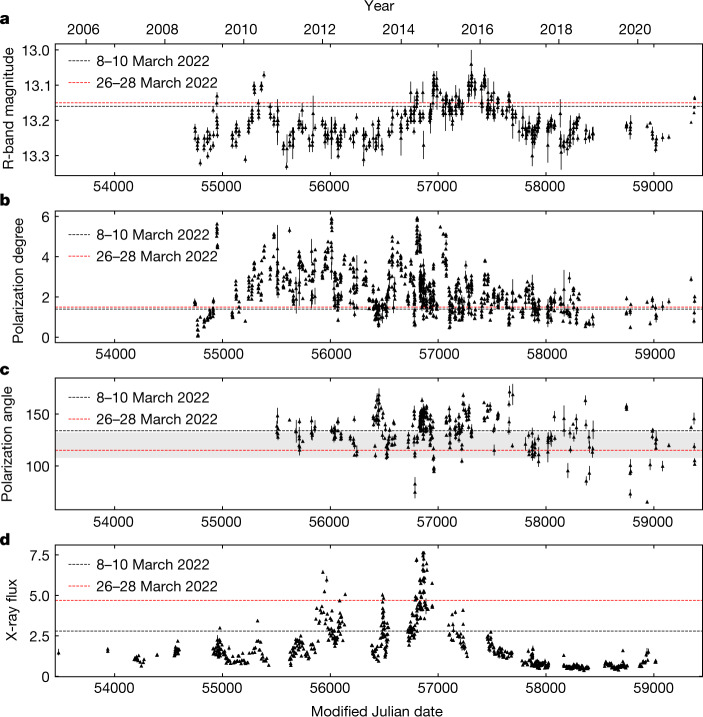
Multiwavelength and polarization archival observations of Mrk 501. **a**–**d**, Optical brightness (R-band, **a**), observed optical *Π* in per cent (**b**), observed optical *ψ* in degrees (**c**) and X-ray flux in ×10^−10^ erg s^−1^ cm^−^^2^ (**d**). The black and red dashed lines indicate the level of the source during the 8–10 March and 26–28 March 2022 IXPE observations, respectively. The grey shaded area in **c** shows the direction of the jet axis. In all panels, the error bars denote the 68% CI.

The first IXPE observation of Mrk 501 took place during the period 8–10 March 2022 (100 ks, MJD 59646–59648) and was accompanied by observations across the electromagnetic spectrum from multiple observatories (Methods). IXPE measured a polarization degree of *Π*_X_ = 10 ± 2% and an electric vector position angle *ψ*_X_ = 134 ± 5^ο^ (measured east of north) over the X-ray energy range of 2–8 keV. Contemporaneous millimetre-radio and optical observations (Extended Data Table 2) measured the degree of polarization *Π*_R_ = 1.5 ± 0.5% along a radio polarization angle *ψ*_R_ = 152 ± 10^ο^ and *Π*_O_ = 4 ± 1% along an optical polarization angle *ψ*_O_ = 119 ± 9^ο^, respectively. A second IXPE observation took place during the period 26–28 March 2022 (86 ks, MJD 59664–59667) yielding *Π*_X_ = 11 ± 2% along *ψ*_X_ = 115 ± 4^ο^. Simultaneously to the second observation, the optical polarization was measured as Π_O_ = 5 ± 1% along *ψ*_O_ = 117 ± 3^ο^(Extended Data Table 3). The two observed *ψ*_X_ are consistent within 3*σ*. The radio and optical *ψ* also lie within 3*σ* from each other and *ψ*_X_. Moreover, the position angle of the jet of Mrk 501 has been determined through Very Long Baseline Array imaging at 43 GHz to be 120 ± 12^ο^(ref. ^22^). This would suggest that, in both cases, radio-to-X-ray *ψ* is aligned with the jet axis within uncertainties (Fig. 1). We do not find evidence of polarization variability during either IXPE observation. Compared with the archival multiwavelength observations, we find the flux and polarization of Mrk 501 for both observations to be within one standard deviation of the median of the long-term light curves (Fig. 2). Blazars such as Mrk 501 are known to reach X-ray fluxes during outbursts as much as an order of magnitude higher. For the first IXPE observation the measured X-ray flux indicates an average activity state, whereas during the second observation we find evidence of a slightly elevated X-ray flux state. Compared with the historical maximum X-ray flux, during our observations Mrk 501 was a factor of three and a factor of two fainter, respectively.

**Fig. 3:**
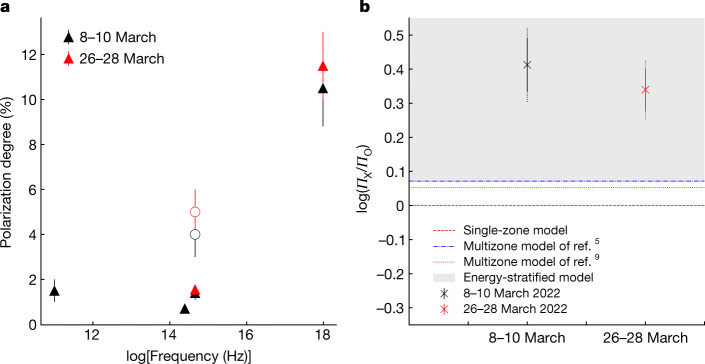
Multiwavelength polarization of Mrk 501. **a**, Multiwavelength polarization degree of Mrk 501 from radio to X-rays. Black symbols are for the 8–10 March observation and red for the 26–28 March observation. The open symbols show the host-galaxy corrected-intrinsic optical polarization degree. **b**, Comparison between the observed logarithm of the X-ray and optical *Π* ratio and the expectations from single-zone model (red dashed line), two turbulent multizone jet models (dash-dotted blue and dotted magenta lines) and energy-stratified models (grey shaded area) for both IXPE observations (black for 8–10 March and red for 26–28 March). The solid error bars show the ratio uncertainty from the IXPE measurements; the dotted error bars show the full uncertainty including optical uncertainties. In both panels, the error bars denote the 68% CI.

The polarization measurements reported here reveal an increase in *Π* towards higher frequencies, with a degree of X-ray polarization that is more than twice the optical value (Fig. 3). This is in tension with the single-zone, turbulent multizone and magnetic reconnection models discussed above. There is no significant variability within the duration of the individual IXPE observations, in contrast to the predicted behaviour if turbulent cells moved in and out of the emission region on timescales of less than 2 days. On the other hand, the low (<10%) optical and X-ray polarization suggests significant disordering of the local magnetic field, possibly due to the presence of stationary turbulence. The wavelength dependence and lack of variability of *Π*, plus the constancy of *ψ* and its alignment with the jet direction, supports the shock-accelerated energy-stratified electron population scenario^4,19,21^. Previous intensely sampled measurements of the polarization of Mrk 501 have found variations in *Π*_O_ by ±5% and in *ψ*_O_ by 50^ο^ from one night to the next^10^. These apparently discrepant results can be reconciled if the turbulence of the plasma flowing through shocks in the jet is only intermittent, as has been found previously in other blazars^23^. One would also expect deviations of the observed *ψ* from the jet axis as one moves further away from the shock front into more turbulent regions of the jet. At present, the large *ψ* uncertainties prevent us from confirming such behaviour. Future observations of Mrk 501 or similar blazars will allow us to explore the jet’s multiwavelength polarization variability. A prediction of the energy-stratified model is that the X-ray polarization angle of blazars that have synchrotron spectral energy distribution peaks at X-ray frequencies, like Mrk 501, will exhibit rotations^24^.

Probing the magnetic environment of the site of energization of radiating particles has supplied a new method for discriminating among particle acceleration mechanisms in astrophysical jets. The new X-ray polarization observations, in combination with the previously available radio and optical polarization diagnostics, have provided a discriminating set of evidence. Our results demonstrate how multiwavelength polarization uniquely probes the physical conditions in supermassive black-hole systems. Future monitoring of the time variability of multiwavelength polarization with IXPE and other instruments will improve the definition of the range of physical conditions that occur in astrophysical jets.

## Methods

### X-ray polarization observations

IXPE is a joint mission of the US National Aeronautics and Space Administration and the Italian Space Agency (Agenzia Spaziale Italiana). A description of the spacecraft and of the payload is given in ref. ^7^ and the detector units are described in ref. ^25^. Mrk 501 was observed with IXPE over an effective exposure time of 100 ks from 8 to 10 March 2022 (MJD 59646–59648) and again from 26–28 March 2022 (MJD 59664–59666) for 86 ks. The exposure times were selected on the basis of the results of ref. ^26^, which determined that a 100 ks exposure would be sufficient to measure polarization in Mrk 501 in a blind survey. At the approximately 30” angular resolution of IXPE, Mrk 501 is essentially a point source.

**Extended Data Fig. 1:**
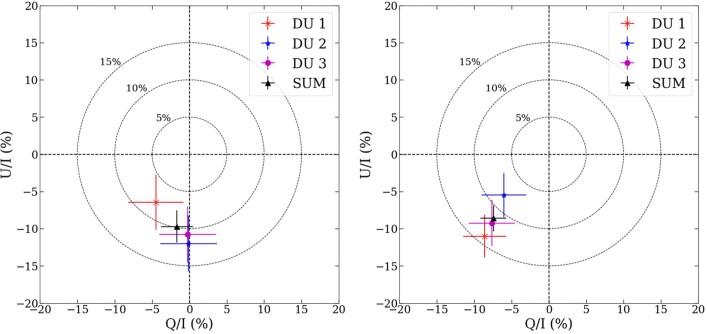
Stokes Q/I and Stokes U/I parameters of our *IXPE* observations during 8–10 March 2022 (left) and 26–28 March 2022 (right). The measurements are shown for the three detectors (DU1 [red x], DU2 [blue star], DU3 [magenta circle]) separately and combined (black triangle). In both panels error bars denote the 68% CI.

The IXPE raw (level-1) data were first reduced and corrected for instrumental polarization artifacts as well as boom and spacecraft motion to create level-2 event files (L2). The L2 data were then corrected for the energy scaling of the detector and bad aspect time intervals following standard procedures within the latest version of the ixpeobssim pipeline^27,28^. The IXPE L2 files contain the polarization information in the form of photon-by-photon Stokes parameters. All the quoted results refer to the average of the three identical IXPE detector units. We selected source photons using xpselect and a circular region with a radius of 60” centred on the source. The polarization degree and angle were determined in the 2–8 keV energy range using three different analysis techniques performed by five independent groups to ensure an unbiased estimation. Those techniques were a model-independent analysis, a spectropolarimetric fit in the X-ray spectral fitting package (XSPEC) and a maximum likelihood spectropolarimetric fit implemented within the MULTINEST algorithm. Although the effect of the photoelectric absorption is negligible over the 2–8 keV energy range of IXPE, the spectropolarimetric fits included photoelectric absorption based on the measured Galactic neutral hydrogen column density toward Mrk 501 of *N*_H_ = 1.69 × 10^20^ cm^−2^ (ref. ^29^). The model-independent analysis applies the formalism of ref. ^30^to a user-defined subset of photons and determines the total Stokes parameters. We have performed both a weighted and unweighted analysis. In the model-independent analysis we do not perform background subtraction. We found that the sky background counts for a 60” region are only 3% of the total counts. We have verified that for a bright blazar such as Mrk 501 the background has a negligible effect on the polarization analysis. For the spectropolarimetric fits, we simultaneously fit 3× *I*, *Q* and *U* spectra (one set from each IXPE detector unit). In XSPEC, following the approach of ref. ^31^, we used an absorbed single power-law component with constant *Π* and *ψ* (CONSTPOL model). For the maximum likelihood spectropolarimetric fit, we used a single power-law spectral component with constant intrinsic *Q* and *U* values. Given the exposure time and flux of Mrk 501 at the time of the IXPE observations, the minimum degree of detectable polarization at a 99% confidence level (MDP99) that we were able to achieve was 6.6% for the 8–10 March and 5.2% for the 26–28 March observations. The source was brighter in X-rays during the 26–28 March observation (see below), hence the lower MDP99. The derived Π and *ψ* values for the different methods are summarized in Extended Data Table 1 for both observations. In both cases, all the measurements through the different analyses are consistent within the uncertainties with the median linear X-ray *Π* and *ψ* of *Π*_X_ = 10 ± 2%, *ψ*_X_ = 134 ± 5^ο^ and *Π*_X_ = 11 ± 2% and *ψ*_X_ = 115 ± 4^ο^, respectively. Extended Data Figure 1 shows the Stokes *Q*/*I* and Stokes *U*/*I* values of our observations along with the MDP99. Depending on the emission model, variability timescales are expected to range from subday to a few days^18^. A 16-day interval between observations allows us to look for variability on a timescale of a few days, which, however, we do not find. We have also searched for variability within the individual IXPE observations. This was done by splitting the IXPE exposures into two and three equal-sized time bins. We again do not find evidence for variability within the uncertainties.

### Multiwavelength observations

Here we report on a subset of our contemporaneous multiwavelength campaign from radio to TeV γ-rays, which is summarized in Extended Data Tables 2 and 3 and Fig. 3. The complete multiwavelength dataset will be presented in a forthcoming paper.

### Millimetre-radio observations

Polarimetric millimetre-radio measurements at 3.5 mm (86.24 GHz) and 1.3 mm (230 GHz) were obtained with the 30 m Telescope of the Institut de Radioastronomie Millimetrique (IRAM), located at the Pico Veleta Observatory (Sierra Nevada, Granada, Spain), on 9–10 March 2022 (MJD 59647–59649), within the Polarimetric Monitoring of AGN at Millimeter Wavelengths (POLAMI) programme, http://polami.iaa.es/ (refs. ^32–34^). Weather-related reasons prevented us from obtaining radio observations during the second IXPE exposure. Under the POLAMI observing setup, the four Stokes parameters (*I*, *Q*, *U* and *V*) are recorded simultaneously using the XPOL procedure^35^. The data reduction, calibration and managing and flagging procedures used in POLAMI are thoroughly described in ref. ^32^. The source was relatively stable in flux during the observations at both 1.3 and 3.5 mm with total flux densities of 0.71 ± 0.04 Jy and 0.73 ± 0.04 Jy at 3.5 mm and 0.41 ± 0.02 Jy and 0.39 ± 0.02 Jy at 1.3 mm, on 9 and 10 of March, respectively. Also, the polarized flux at 3.5 mm remained stable both in the linear polarization degree and the angle between the two dates. No polarization above 3.46% (95% confidence upper limit) was detected at 1.3 mm.

### Optical and infrared observations

Optical polarization observations were performed using several telescopes across the world: the Nordic Optical Telescope on the night of 8–9 March (MJD 59647); the Tohoku 60 cm (T60) telescope at the Haleakala Observatory on 10 March (MJD 59649) and on 28 March (MJD 59667); the 2.2 m Calar Alto Observatory and 1.5 m Sierra Nevada Observatory telescopes on 8–10 March; the AZT-8 telescope of the Crimean Astrophysical Observatory and the St Petersburg State University LX-200 telescope during the periods 8–10 March and 25–28 March 2022.

The Nordic Optical Telescope observations used the Alhambra Faint Object Spectrograph and Camera (ALFOSC) in four bands (BVRI) in the standard polarimetric mode. The data were then analysed with the semi-automatic pipeline developed at the Tuorla Observatory using standard photometric procedures^36,37^. Both highly polarized and unpolarized standard stars were observed during the same night for calibration purposes. The T60 polarimetric measurements were performed using the Dipol-2 polarimeter^38^. Dipol-2 is a remotely operated double-image charged coupled device polarimeter, which is capable of recording polarized images in three (BVR) filters simultaneously^39–42^. We obtained 24 individual measurements of the Stokes *Q*/*I* and *U*/*I* parameters simultaneously in three filters (BVR). Twenty unpolarized and two highly polarized (HD204827 and HD25443) nearby standard stars were observed for calibration and determination of the polarization angle zero point. The individual measurements were used to compute nightly average values using the ‘2× sigma-weighting algorithm’. The algorithm iteratively filters out outliers, assigning smaller weights to these measurements. The errors on the Stokes *Q*/*I* and *U*/*I* parameters were computed as standard errors of the weighted means. These errors were then used to estimate uncertainties on the polarization degree and angle^42,43^. The Calar Alto Observatory observations were performed in the Johnson Cousins R_c_ optical band by the Calar Alto Faint Object Spectrograph in imaging polarimetric mode on the 2.2 m telescope. The data were reduced following standard analysis procedures using both unpolarized and polarized standard stars for calibration purposes. Similarly, Mrk 501 was observed by the 1.5 m telescope at Sierra Nevada Observatory using polarized R_c_ filters during the three nights. The 70 cm AZT-8 telescope and the 40 cm LX-200 telescope observations were carried out in the Cousins R-band. Both telescopes are equipped with nearly identical imaging photometers–polarimeters based on a ST-7 camera. Two Savart plates rotated by 45^o^ relative to each other are swapped to measure the relative Stokes *q* and *u* parameters from the two split images of each source in the field. The polarization parameters for each observation are produced by the sum of 15 × 30 s consecutive exposures. The data are then corrected for bias, flat field and background level, and calibrated for instrumental and interstellar polarization using the (assumed) unpolarized comparison stars 1, 4 and 6 from ref. ^44^. The same stars were used to perform differential photometry. During both IXPE observations, all the optical polarization observations are within uncertainties, which suggests no significant variability.

Observations were also obtained with the WIRC+Pol instrument^45^ on the 200-inch Palomar Hale telescope in the J-band. WIRC+Pol uses a polarizing grating to disperse the light into four beams that sense the four different components of linear polarization (0^ο^, 45^ο^, 90^ο^, 135^ο^), and a half-wave plate for beam swapping to improve polarimetric sensitivity^46,47^. Data reduction made use of the WIRC+Pol Data Reduction Pipeline software (https://github.com/WIRC-Pol/wirc_drp(ref. ^45^)). The pipeline software averages the measurements over the course of the half-wave plate rotation cycles to account for subtle differences in light paths through the instrument, and reports the degree and angle of polarization in each band. The results were verified with the use of both polarized and unpolarized standard stars. For additional details on the data reduction, see ref. ^48^.

The starlight from the host galaxy (assumed to be unpolarized) of Mrk 501 contributes a significant fraction of the optical flux. For this reason, the observed *Π*_O_ needs to be corrected for the depolarization effect of the host galaxy. To achieve this, we need to estimate the contribution of the host galaxy (*I*_host_, in mJy) within the aperture used for the analysis of individual observations. The light profile of Mrk 501's host galaxy has been fully characterized in the R-band in ref. ^49^. This allows us to estimate *I*_host_for each observation separately. We then subtract *I*_host_from the total intensity *I* and estimate the intrinsic polarization degree following ref. ^36^ as *Π*_intr _= *Π*_O_ × *I*/(*I* − *I*_host_). Owing to the Dipol-2 instrument layout as well as the lack of a light profile model for the host galaxy in the J-band we are not able to accurately estimate the host-galaxy contribution to the polarization measurements for the T60 and Palomar Hale telescopes. For this reason, the measurements from T60 and Hale should be treated as lower limits to the intrinsic polarization degree. For the remaining telescopes, we calculate *Π*_intr_in the R-band for each observation and then estimate a median. We find the median intrinsic polarization degree and its uncertainty to be *Π*_intr_ = 4 ± 1% for the 8–10 March observation and *Π*_intr_ = 5 ± 1% for the 26–28 March observation. Figure 3 shows the multiwavelength polarization degree from radio to X-rays.

### X-ray observations

During the IXPE observations we independently measured the X-ray total flux and spectrum with the X-Ray Telescope^50^ on the orbiting Neil Gehrels Swift Observatory (Swift) in Window Timing mode (WT, 4 × 1 ks exposures, with 2 × 1 ks for each IXPE observation) and with the Nuclear Spectroscopic Telescope Array (NuSTAR, 20 ks exposure^51^) during the 8–10 March observation. We extracted the X-ray spectrum from each telescope following standard analysis procedures and the latest calibration data files. For the source regions we used a circular radius of 47” and 49” for Swift and NuSTAR, respectively. To estimate the background for the NuSTAR spectra we used a 147’’ circular region outside of the region containing significant photon counts from Mrk 501. The background for Swift was extracted using the same size circular region from an available blank sky WT observation from the Swift archive. For the 8–10 March observation, we fit the combined Swift and NuSTAR data in XSPEC with an absorbed log-parabola model *N*(*E*) = (*E*/*E*_p_)^(−*α* − *β*log(*E*/*E*p))^, in the 0.3–79 keV energy range; *N*(*E*) is the number of photons as a function of energy *E*. *N*_H_ was set to the Galactic value and the pivot energy was set to *E*_p_ = 5 keV. This model provides a reasonably good fit to the data (*χ*^2^/dof = 862/850) with best-fit parameters *α* = 2.27 ± 0.01 and *β* = 0.28 ± 0.01. We also tested a single power-law model; however, there is clear curvature in the spectrum and the fit is statistically worse (*χ*^2^/dof = 2,005/851). We measure the flux of the source in the 2–8 keV range to be (10.0 ± 0.5) × 10^−11^ erg s^−1^ cm^−^^2^. We do not find evidence for variability during the IXPE observations. For the 26–28 March observation we follow the same procedure using only the available Swift data. The source was in a higher flux state with *α* = 2.05 ± 0.02 and *β* = 0.26  ±0.04 and flux in the 2–8 keV range of (21.0 ± 0.6) × 10^−11^ erg s^−1^ cm^−^^2^. The Swift observations show a change from 12 to 14 counts s^−1^ (17% increase) from the beginning until the end of the IXPE observation. The results from our multiwavelength campaign are summarized in Extended Data Tables 2 and 3.

### Activity state of Mrk 501

Mrk 501 is a BL Lac object at a redshift of *z* = 0.033, corresponding to a luminosity distance of 141.3 Mpc, assuming a flat Lambda cold dark matter (ΛCDM) cosmological model with a matter density *Ω*_m_ = 0.27 and a Hubble constant *H*_0_ = 71 km s^−1^ Mpc^−1^ (ref. ^52^), and a synchrotron peak frequency *ν*_syn _~ 2.8 × 10^15^ Hz (ref. ^53^). It is among the brightest sources in the sky at very high γ-ray energies (>0.1 Tev), and is well-studied across the electromagnetic spectrum^54–58^. We use archival data from Swift (https://www.swift.psu.edu/monitoring/), the Steward observatory (http://james.as.arizona.edu/~psmith/Fermi/(ref. ^59^)), the RoboPol programme (http://robopol.physics.uoc.gr/(ref. ^3^)) and the Boston University blazar monitoring programme (https://www.bu.edu/blazars/index.html) to build the long-term light curves of Mrk 501 in optical brightness (R-band magnitude), optical polarization degree, polarization angle and X-ray flux (Fig. 2). The optical observations cover a range from October 2008 to June 2021. For the R-band, the source varied between 13.53 min and 13.24 min, with a median of 13.4 min. The median observed *Π*_O_ (not corrected for the host-galaxy contribution) was 2.1% with a minimum of 0.07% and a maximum of 5.9%. The *ψ*_Ο_ typically fluctuates about the jet axis (120 ± 12^ο^) with a median of 136^ο^, and a minimum and maximum of 65^ο^ and 171^ο^, respectively. The X-ray observations cover a range from April 2005 to June 2020. The median X-ray flux in the 0.3–10 keV was 15 × 10^−11^ erg s^−1^ cm^−^^2^, with a minimum and maximum at around 3.7 × 10^−11^ erg s^−1^ cm^−^^2^ and 76 × 10^−11^ erg s^−1^ cm^−^^2^, respectively. At the time of the IXPE observations our multiwavelength campaign finds the flux and polarization of the source within one standard deviation of the median of the respective archival light curve. For the first IXPE observation the X-ray flux of the source seems to correspond to an average state, whereas in the second observation we find the source in a slightly elevated flux state.

## Online content

Any methods, additional references, Nature Research reporting summaries, source data, extended data, supplementary information, acknowledgements, peer review information; details of author contributions and competing interests; and statements of data and code availability are available at 10.1038/s41586-022-05338-0.

## Figures and Tables

**Extended Data Table 1:**
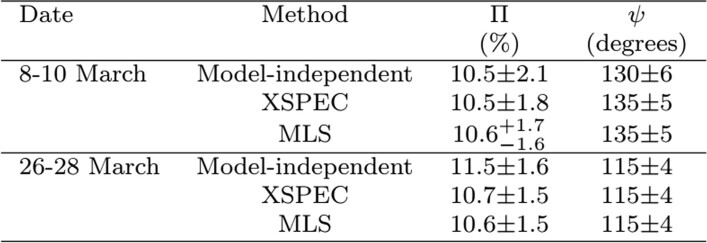
Median polarization degree and angle measurements from the *IXPE* data analysis performed by independent groups using three analysis techniques

**Extended Data Table 2:**
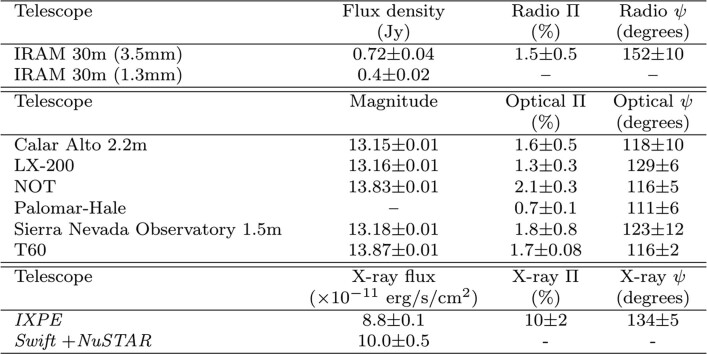
Multiwavelenth and polarization observations for the 2022 March 8–10 observation

*Table comments*: The millimeter-radio flux density is in Janskys. For the millimeter-radio and optical observations we report the median estimate of the observations during the *IXPE* observation. The listed uncertainty is either the standard deviation of the measurements or the median uncertainty, whichever is larger. For the NOT and T60 analysis we used a circular 1.5” radius aperture. For the data analysis of remaining optical telescopes we used a 7.5’ aperture. The Palomar observations are in the J-band. *ψ* is given in degrees. The X-ray fluxes are estimated in the 2–8 keV range, and given in units of 10^−11^ erg/s/cm^2^.

**Extended Data Table 3:**
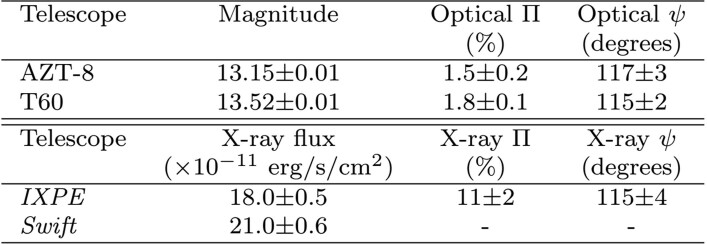
Multiwavelenth and polarization observations for the 2022 March 26–28 observation

*Table comments*: Same as in Extended Data Table 2.

## Data Availability

The data that support the findings of this study are either publicly available at the HEASARC database or available from the corresponding author upon request.
